# Adjunctive anifrolumab for recalcitrant discoid lupus erythematosus in the setting of systemic lupus erythematosus: A case report

**DOI:** 10.1016/j.jdcr.2025.02.024

**Published:** 2025-03-11

**Authors:** Gabriela Soto-Canetti, Jordan Talia

**Affiliations:** a Department of Dermatology, Icahn School of Medicine at Mount Sinai, New York, New York; b Ponce Health Sciences University School of Medicine, Ponce, Puerto Rico

**Keywords:** anifrolumab, discoid lupus erythematosus, interferon-1, scarring alopecia, skin of color, systemic lupus erythematosus

## Introduction

Discoid lupus erythematosus (DLE) is a subtype of cutaneous lupus erythematosus (CLE) and often associated with systemic lupus erythematosus (SLE). The characteristic scarring and depigmentation of DLE adds to the disease burden brought on by SLE and is known to substantially impact the quality of life of patients.^1^ Current accepted therapies for DLE include topical and intralesional corticosteroids, topical calcineurin inhibitors, antimalarials, immunosuppressants, systemic corticosteroids, thalidomide, and topical retinoids.^1^ However, recalcitrant cases of CLE have shown variable responses to traditional treatment options and targeted, nonsteroidal alternatives, such as biological agents, have been suggested in recent years. Anifrolumab is a human monoclonal antibody directed against the type I interferon (IFN-1) receptor subunit 1 that was approved by the US Food and Drug Administration for SLE in 2021.^2^ The IFN-1 pathway has been implicated in the pathogenesis of SLE and CLE with some patients demonstrating elevated serum IFN-1 levels.^2^ The second phase 3 trial on anifrolumab for SLE (type I interferon inhibitor anifrolumab in active systemic lupus erythematosus [TULIP-2]) demonstrated higher rates in reduction of glucocorticoid use and decreased severity of skin disease when compared with placebo.^3^ We present the case of a patient with severe refractory DLE who achieved significant clearance of skin disease after treatment with adjunctive anifrolumab.

## Case presentation

**Figs 1-3:**
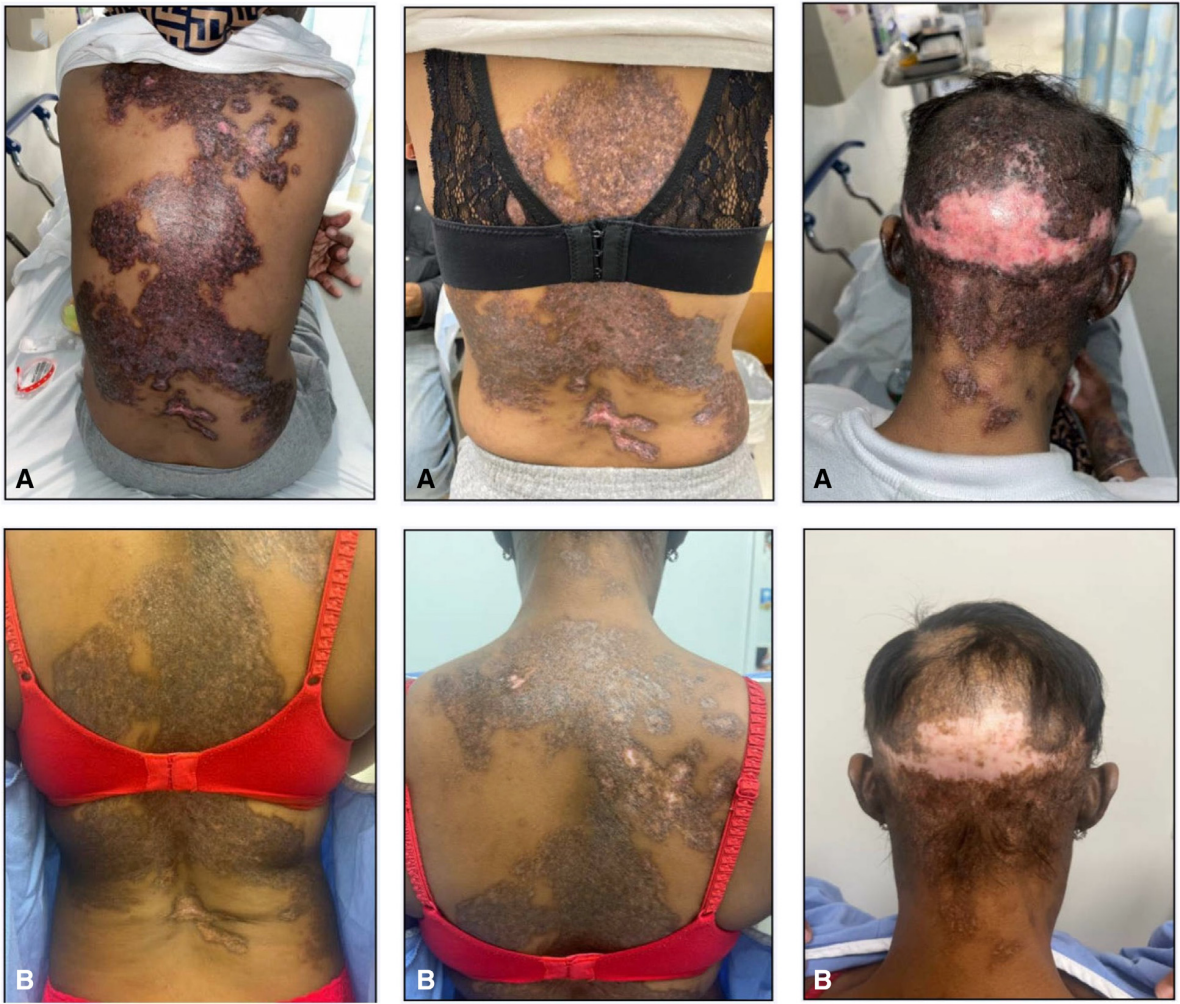
Physical examination shows scattered dyspigmented plaques with areas of scarring in a generalized distribution over the back (1**A**, 2**A**) and scalp with diffuse scarring alopecia (3**A**). Results after 5 months of anifrolumab infusion showing healing of previously affected patches and postinflammatory hyperpigmentation (1**B**-3**B**).

A 35-year-old woman with a 15-year history of SLE with positive antinuclear antibodies presented to our clinic with a flare of cutaneous symptoms and associated general malaise. The patient had a previous history of malar and discoid rash, arthritis, pleuropericarditis, Class III and V lupus nephritis, idiopathic thrombocytopenic purpura, myopericarditis, and pulmonary hypertension. She is also a current tobacco smoker. Physical examination revealed scattered dyspigmented plaques with areas of scarring and in a generalized distribution over the face, chest, back, extremities, and scalp with diffuse scarring alopecia (Figs 1, *1A-3A*). Previous treatments included hydroxychloroquine, mycophenolate mofetil, azathioprine, methotrexate, rituximab, and belimumab with intermittent usage of high-dose corticosteroids, all of which had been ineffective or not tolerated. The patient presented after having self-discontinued all treatment 6 months earlier, citing perceived ineffectiveness of recent medications. Her most recent therapy included mycophenolate mofetil 500 mg twice daily, prednisone 40 mg daily, and hydroxychloroquine 200 mg daily. At the time of presentation, she was treated with tacrolimus ointment, with minor improvement in cutaneous lesions. In March 2024, treatment with anifrolumab 300 mg infusions every 4 weeks was initiated in combination with mycophenolate mofetil 500 mg twice daily, hydroxychloroquine 200 mg daily, and prednisone 20 mg daily. Five months after initiation of anifrolumab, there were no new cutaneous lesions and significant healing of previously affected patches, as well as some improvement of hair loss (Figs 1, *1B-3B*). Additionally, the patient reported improvement in arthritis, which was associated with her active SLE. At the time of this writing, the patient was on anifrolumab, mycophenolate mofetil, hydroxychloroquine, and started on a prednisone 20 mg taper. No adverse effects were reported during the transitional and follow-up periods.

## Discussion

Recalcitrant CLE has proven a challenge due to heterogeneous presentations and resistance to traditional treatment modalities. In recent years, monoclonal antibodies have been studied as potential alternatives for treating CLE, with belimumab as the first US Food and Drug Administration-approved biologic for SLE.^1^ However, no medications have been approved by the US Food and Drug Administration for the treatment of CLE specifically. The IFN-1 pathway plays a major role in the pathogenesis of CLE and SLE, resulting in activation and increased survival of dendritic, B- and T-lymphocytes.^1^ The TULIP-2 trial, a second phase 3 trial on anifrolumab for SLE, demonstrated superiority of anifrolumab administration to placebo in reduction of glucocorticoid use and reduction in severity of skin disease over 52 weeks.^3^ Among patients with at least moderately active skin disease, there was a reduction of 50% or more in CLE Disease Area and Severity Index at week 12 in 49% of the patients with anifrolumab compared with 25% of patients receiving placebo.^3^ Of note, the TULIP-2 trial reported that the addition of anifrolumab to standard of care of regimens resulted in an increased incidence of herpes zoster and upper respiratory tract infections and only one death from pneumonia among the anifrolumab group.^3^

Although anifrolumab has only been approved for SLE, isolated clinical experiences have also reported adequate control of cutaneous disease without severe adverse effects.^1^^,^^4^ Günther et al^5^ reported a decline in Cutaneous Lupus Erythematosus Disease Area and Severity Index activity score paralleled with a decreased blood interferon score in 7 patients with refractory CLE who were treated with anifrolumab. In total, 20 publications reporting on 78 patients have demonstrated rapid cutaneous symptom improvement with anifrolumab therapy.^6^ Thus anifrolumab represents a potentially efficacious treatment alternative for patients with recalcitrant DLE without the risks brought on by immunosuppressant therapy. An additional benefit includes the potential for greater medication adherence. A limitation to anifrolumab therapy may be in its use for patients with SLE, as anifrolumab has been shown to have limitations in lupus nephritis.^7^ Patients with systemic disease may require higher dosing or additional therapy to anifrolumab. Our patient had a significant improvement in the appearance and severity of cutaneous lesions, as well as some improvement in alopecia, after 5 months on adjunctive anifrolumab. Previous treatment with antimalarials, rituximab, azathioprine, methotrexate, mycophenolate mofetil, and belimumab were ineffective, intolerable, or presented with adherence difficulties. Three cases have been previously reported demonstrating significant improvement in CLE-associated patchy alopecia, as well as improvement in mucocutaneous manifestations of SLE, favoring a potential anti-inflammatory effect of anifrolumab.^8^, ^9^, ^10^ Furthermore, an ongoing phase 3 clinical trial of subcutaneous anifrolumab for CLE has the potential to enhance the growing peer-reviewed data that is currently available (NCT06015737). Additional multicenter studies may be instrumental in adequately characterizing the effect of anifrolumab on DLE-associated scarring alopecia, potentially expanding its indication as a treatment alternative for chronic CLE, particularly in the setting of SLE.

## Conflicts of interest

Dr Talia has served as a consultant for Leo Pharma, UCB, Calliditas Therapeutics, Primus Pharmaceuticals, Stifel Financial, Arcutis Biotherapeutics, and Johnson & Johnson. Author Soto-Canetti has no conflicts of interest to declare.
